# The Efficacy of Povidone-Iodine in Eradicating Staphylococcus aureus Biofilm on Stainless Steel Alloy Implants

**DOI:** 10.5704/MOJ.2603.002

**Published:** 2026-03

**Authors:** AA Sofian, F Che-Hamzah, NA Khirul-Ashar, MF Noorman, AA Ab-Halim, S Amin-Nordin, NM Sither-Joseph

**Affiliations:** 1 Department of Orthopaedics and Traumatology, Universiti Teknologi MARA, Sungai Buloh, Malaysia; 2 Department of Orthopaedics, Universiti Putra Malaysia, Serdang, Malaysia; 3 Department of Medical Microbiology, Universiti Putra Malaysia, Serdang, Malaysia

**Keywords:** biofilm, povidone-iodine, implant-related infection, Staphylococcus aureus

## Abstract

**Introduction:**

*Staphylococcus aureus* is the leading biofilm-forming microorganisms in orthopaedic implant infections. The biofilms formed are difficult to eradicate and resistance to antibiotics. This current study aims to determine the effectiveness of povidone-iodine; an antiseptic solution in eradicating *S. aureus* biofilm on stainless steel alloy. In addition to the usual Colony-Forming Unit (CFU) used for verification, Scanning Electron Microscope (SEM) is used to validate the formation and eradication of the biofilms.

**Materials and methods:**

This is an *in vitro* study where the biofilm is formed by inoculating clinically isolated *S. aureus*, incubated for 24 hours onto stainless steel alloy 316L implants. The implants are then irrigated using povidone-iodine solution with varying concentrations (5 and 10%) and durations (30, 60, and 180 seconds). The anti-biofilm effect was evaluated using plating and SEM methods to confirm its effectiveness. The process is repeated after 24 hours of post-irrigation reincubation to detect any rebound growth.

**Results:**

No biofilm seen after irrigation with povidone-iodine at 5% and 10% concentrations at 30, 60 and 180 seconds, respectively, in both CFU count and SEM. This result is replicated after 24 hours of reincubation, in assessing for rebound growth.

**Conclusion:**

Our study supports that a minimum of 5% povidone-iodine with a minimum irrigation time of 30 seconds are effective at eliminating *S. aureus* biofilm on stainless steel alloy implants. Both CFU count and SEM yield similar value in validating the presence of biofilm. Additionally, SEM allows visualisation of the morphology of the biofilm.

## Introduction

Orthopaedic implants such as internal fracture-fixation devices and joint prostheses, are increasingly used today^1-3^. Despite the success of these devices, their susceptibility to infection remains a problem. Implant-related infection can occur despite strict preventive measures, such as the administration of antibiotic prophylaxis, the use of antiseptic scrub, maintaining ultra-clean operation theatre environment and careful patient selection. The risk varies between 0.4% and up to 16.1% depending on the type of fracture, ranging from close to varying degrees of open fracture^4,5^. Once an infection occurs, treatment becomes a significant challenge.

One of the culprits hindering treatment success is the presence of surface-adhering microorganisms forming a biofilm. A biofilm is a community of microorganisms that adhere to either inert or living surfaces, encapsulated within a self-produced polymeric matrix. Biofilms can develop on both biotic and abiotic surfaces, occurring in natural environments as well as healthcare settings^2^. The formation of biofilms on implants causes three major problems. Firstly, bacterial communities on these surfaces represent a reservoir of bacteria that can be shed into the body, leading to a persistent infection. Secondly, biofilm bacteria are highly resistant to antibiotic treatment; therefore, once these bacterial communities form, they become extremely difficult to eliminate with conventional antimicrobial therapies. Finally, because host responses and antimicrobial therapies are often cannot eliminate bacteria growing in a biofilm, a chronic inflammatory response at the site of the biofilm may be produced^3^.

A significant proportion of implant-related infections are caused by *Staphylococcus spp.*, particularly *Staphylococcus epidermidis* and *Staphylococcus aureus*, with the latter being the predominant culprit^6,7^. Together, these two staphylococcal species account for two-thirds of infection isolates^8^. They are the primary causative agents in orthopaedic infections, presenting major treatment challenges due to their ability to form small colonies and develop biofilms^8,9^. Among the two, *Staphylococcus aureus* is known for its increased virulence, production of various toxins and virulence factors, and the ability to produce stronger biofilms. It is the focus of this study due to its considerable clinical impact, while *Staphylococcus epidermidis* is less virulent but includes more antibiotic-resistant strains^10^.

The mainstay treatment for implant-related infection is antibiotic therapy. However, relying solely on antibiotic therapy may not be sufficient. Therefore, the management should include surgical intervention, such as debridement with or without the removal of the implant. According to conventional practice, the removal of implants was deemed necessary for the complete eradication of biofilm bacteria. However, newer concepts challenge this dogma. After a confirmed microbiological diagnosis, and with the rapid administration of appropriate antibiotics, many orthopaedic devices can be retained in place following thorough surgical debridement^11^.

Several studies have explored the most effective methods for eradicating biofilms using irrigation. Smith *et al*^12^ demonstrated that irrigation and debridement of an infected prosthetic joint infection model using 4% chlorhexidine gluconate solution was effective in treating methicillin-resistant *S. aureus* biofilm, even at concentrations as low as 2%. In a study by Anglen *et al*^13^ which focuses on S. epidermidis biofilm on stainless steel screws, it was found that (1) power irrigation was more effective than a bulb syringe at bacterial removal, (2) antibiotic-impregnated normal saline irrigation was no more effective than normal saline alone, and (3) castile soap (a detergent) irrigation was more effective than normal saline alone in eradicating biofilm. In a separate study, Moussa *et al*^14^ discovered that benzalkonium chloride (a detergent) was effective against biofilms formed by *S. epidermidis*, *S. aureus*, and *Pseudomonas aeruginosa*.

Hosaka *et al*^15^ investigated the antibacterial activity of povidone-iodine against *in vitro* cultured dual species biofilm. The organisms selected for the study were *Porphyromonas gingivalis* and *Fusobacterium nucleatum*. Their conclusion suggested that a 30-second application of 2% povidone-iodine was effective in suppressing biofilms produced by both organisms.

Povidone-iodine is widely utilised in surgical settings, including orthopaedic procedures, for its antiseptic properties and effectiveness against biofilms. These solutions demonstrate broad-spectrum antimicrobial activity against various bacterial and fungal species, as well as certain viruses^16-18^. Its application as an irrigation solution during surgery is supported by various studies and guidelines. The World Health Organization (WHO) and the Centers for Disease Control and Prevention (CDC) recommend the use of aqueous povidone-iodine solutions for incisional wound irrigation to prevent surgical site infections. It is also supported by International Consensus Meeting on Musculoskeletal Infection^19^. These endorsements are based on evidence demonstrating povidone-iodine's broad-spectrum antimicrobial activity and its efficacy in reducing surgical site infections. However, questions persist regarding the effective concentration and duration of povidone-iodine application to eliminate biofilm. To our knowledge, there has been no study specifically addressing the use of povidone-iodine against biofilm on stainless steel alloy implants.

We aim to investigate the efficacy of povidone-iodine in eradicating *S. aureus*-produced biofilm on stainless steel alloy implants to enhance the outcome of debridement in infected implant fixation. Povidone-iodine is well-known for its broad-spectrum antimicrobial activity against bacteria, fungi, and viruses. While its use in surgical site preparation is extensively documented, recent evidence suggests its potential to penetrate and disrupt biofilms. For example, studies by Barreto *et al*^20^ and Bigliardi *et al*^21^ have shown its effectiveness in reducing bacterial biofilm burden in both in vitro and clinical settings. Our goal is to establish guidelines for the optimal concentration and duration of povidone-iodine application during debridement. This research is crucial as it has the potential to reduce frequency of multiple debridement and shorten hospital stays. This could possibly alleviate the economic burden on patients, families, healthcare institutions, and ultimately on the public as a whole. We anticipate that by identifying the optimal time and concentration of povidone-iodine, we can establish standardised guidelines for the debridement of infected orthopaedic implants and prosthetic joint infections.

## Materials And Methods

This experimental *in vitro* study was designed to determine the efficacy of povidone-iodine solution in eradicating *S. aureus* biofilms on stainless steel alloy implants when exposed to clinically relevant concentrations for exposure times commonly encountered in clinical practice. The concentrations under investigation were 5% and 10% concentrated povidone-iodine. The choice of 10% povidone-iodine is based on its standardised use in many hospitals and its widespread adoption by surgeons. Meanwhile, 5% povidone-iodine is considered to be easily prepared intra-operatively by diluting one part of the 10% solution with one part diluent. The durations of povidone-iodine submersion studied were 30, 60, and 180 seconds. The samples were divided into two main groups: Group I (24-hour incubation) and Group II (reincubation). Each group was further subdivided into five categories: A, which served as the positive control; B, the negative control for 5% povidone-iodine; C, the test sample treated with 5% povidone-iodine; D, the negative control for 10% povidone-iodine; and E, the test sample treated with 10% povidone-iodine.

A clinically isolated and confirmed Staphylococcus aureus strain, identified using polymerase chain reaction and Sanger sequencing, was obtained from the Microbiology Lab, Department of Medical Microbiology, Faculty of Medicine and Health Sciences, Universiti Putra Malaysia. *S. aureus* was chosen because it is a common organism associated with orthopaedic implant-related infections and due to its well-documented tendency to form biofilm. The bacteria were suspended in a liquid culture of trypticase soy broth (TSB) and incubated at 37°C for 24 hours in a shaker incubator rotating at 60rpm. Growth in the culture medium was verified by measuring its optical density at 600nm (OD600) using a spectrophotometer [Eppendorf BioPhotometer Plus®].

One-third tubular plates of 3.5mm medical-grade stainless steel 316L implants were utilised. These plates, originally 12-hole configurations, were cut into uniform lengths of 1.5cm. Subsequently, they were packed and sterilised through autoclaving before use.

To prepare Mueller-Hinton agar (MHA), 34.0g of MHA base powder was mixed with 1L of distilled water in a Schott bottle. The bottle was thoroughly shaken to ensure uniform mixing. After autoclaving, the mixture was allowed to cool to 50°C before being poured into petri dishes. Subsequently, it was cooled down and stored in refrigerator at 4°C.

To prepare Trypticase Soy Broth (TSB), 18.0g of TSB base powder was mixed with 500mL of distilled water in a Schott bottle. After autoclaving, the TSB was stored in 50mL centrifuge tubes to prevent contamination.

The povidone-iodine solution was prepared using sterile 10% povidone-iodine. To achieve a 5% concentration, 10mL of autoclaved distilled water was added to 10mL of the 10% povidone-iodine solution.

For the cultivation of biofilms, a 1:40 dilution of the *S. aureus* culture (~1 × 10^8 cells) was inoculated into 2.0mL of TSB on a 12-well polystyrene plate. The plate contained a stainless-steel plate positioned in four quadrants.

The stainless-steel implants underwent incubation at 37°C for a duration of 24 hours with rotation at 60rpm. Subsequently, each implant containing biofilm was extracted and subjected to three washes with 2mL PBS solution to eliminate planktonic bacteria. The cleaned implants were then transferred to a new 12-well polystyrene plate. Biofilm presence on the implants was confirmed through Colony-Forming Units (CFU) counts and observation of a filmy glycocalyx matrix using SEM.

Following 24 hours of bacterial growth on the tested implants, the formed biofilm underwent submersion irrigation with 2mL of povidone-iodine, with distinct time intervals of 30, 60, and 180 seconds for each group. Subsequent to povidone-iodine submersion, each group underwent a thorough wash with PBS to eliminate any residual povidone-iodine.

To assess the effectiveness of submersion irrigation, the examined implants from all the groups were immersed in 2mL of PBS and thoroughly scraped with plate scrapers immediately after povidone-iodine submersion and initial washing with PBS solution. The resulting PBS suspension was homogenised, serially diluted, and then plated onto MHA plates. The plates were incubated at 37°C for 24 hours, and CFU were subsequently calculated.

For further analysis, one sample from each group underwent examination using a Scanning Electron Microscope (SEM), specifically the JSM-IT 100 InTouchScope^TM^. This was done to observe the morphology and structural characteristics of the bacterial biofilm adhered to the stainless-steel implants. The samples were assessed by a microbiologist, and images were captured for additional analysis to confirm the presence of biofilm.

**Fig. 1:**
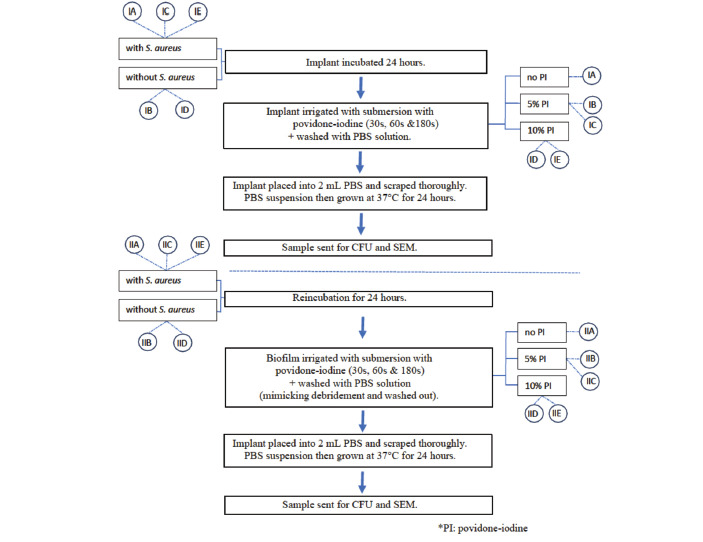
The implant samples were initially divided into two groups (Group I and Group II), each further subdivided into several subgroups. Both groups underwent testing at different time intervals of 30 seconds, 60 seconds, and 180 seconds separately.

Notes: Group I served as the primary testing group to evaluate the effectiveness of povidone- iodine submersion in eradicating S. aureus biofilm. The tested implants were categorised into five subgroups; group IA - S. aureus + no povidone-iodine wash but with PBS wash (positive control), group IB - no S. aureus + 5% povidone-iodine submersion (negative control), group IC - S. aureus + 5% povidone-iodine submersion (triplicate for CFU and one sample for SEM), group ID - no S. aureus + 10% povidone-iodine submersion (negative control), and group IEE- S. aureus + 10% povidone-iodine submersion (triplicate for CFU and one sample for SEM).

Using a new sterile pipette, 0.1mL of the scraped biofilm was aseptically transferred onto MHA plates. The inoculum was evenly spread on the surface of the agar in each plate using a plastic disposable spreader. The agar plates were then incubated at 37°C for 24 hours. After incubation, the colonies on each of the plates were counted (Fig. 1).

Group II was designed to assess bacterial regrowth after submersion with povidone- iodine (post-treatment). Group II continued from Group I, with the addition of a 24-hour reincubation period. This was implemented to simulate real-life situations after debridement and confirm the absence of further growth following submersion in the povidone-iodine solution. The subgroups in Group II are; group IIA - presence of S. aureus + no povidone-iodine wash but with PBS wash (positive control), group IIB - no S. aureus + 5% povidone-iodine submersion (negative control), group IIC - S. aureus + 5% povidone-iodine submersion (triplicate for CFU and one sample for SEM), group IID - no S. aureus + 10% povidone-iodine submersion (negative control), and group IIE - S. aureus + 10% povidone-iodine submersion (triplicate for CFU and one sample for SEM).

## Results

**Fig. 2:**
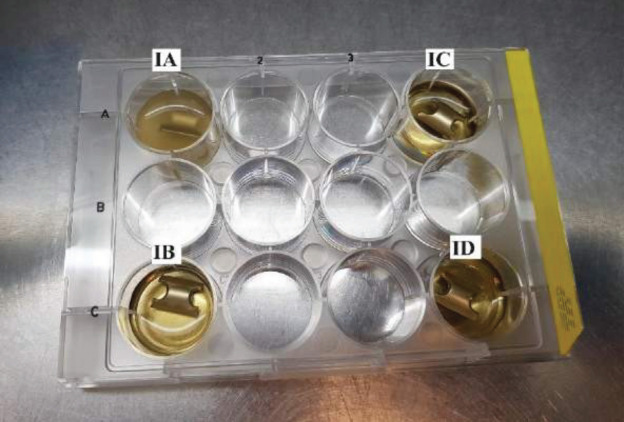
Following the submersion of tested implants, a turbid colour in the PBS solution was observed after 24 hours of incubation in group IA, while clear colouring was noted in groups IB, IC, and ID. Although group IE was not included in the picture, its PBS solution exhibited a clear colour as well.

**Fig. 3:**
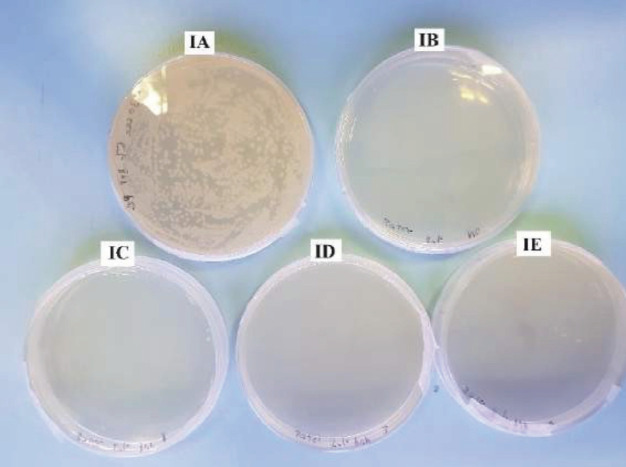
Petri dishes containing Muller-Hinton agar were used for all subgroups with a 30-second irrigation time. Only group 1A exhibited bacterial growth, while the remaining subgroups showed a colony forming unit count of zero.

The CFU count in group IA, where *S. aureus* was present without povidone-iodine washing but subjected to PBS wash (positive control), exhibited an exceedingly high level of bacterial growth that was too numerous to count. In contrast, all other subgroups, whether with or without *S. aureus*, but subjected to povidone-iodine washing, irrespective of concentration, demonstrated no bacterial growth at all during the specified time durations (Fig. 2 and Fig. 3).

Group II aimed to evaluate bacterial regrowth after the completion of the 24-hour testing in Group I. Group IIA exhibited a notably higher CFU count after 24 hours of reincubation. Conversely, all other subgroups, whether with or without *S. aureus*, but subjected to povidone-iodine washing, irrespective of the concentration and submersion time, demonstrated no bacterial growth at all (Table I).

**Table I:** All implants that had been submerged in the povidone-iodine solution showed no growth, irrespective of the concentration of povidone-iodine or the duration of irrigation.

Subgroup		IA			IB			IC			ID			IE		
*S. aureus on implant*		Yes			No			Yes			No			Yes	
Solution	No povidone wash				Washed with 5% povidone-iodine						Washed with 10% povidone-iodine				
Irrigation duration	30s	60s	180s	30s	60s	180s	30s	60s	180s	30s	60s	180s	30s	60s	180s
Sample 1	TNTC	TNTC	TNTC	NG	NG	NG	NG	NG	NG	NG	NG	NG	NG	NG	NG
Sample 2							NG	NG	NG				NG	NG	NG
Sample 3							NG	NG	NG				NG	NG	NG
**Subgroup**		**IIA**			**IIB**			**IIC**			**IID**			**IIE**	
*S. aureus on implant*		Yes			No			Yes			No			Yes	
Solution	No povidone wash				Washed with 5% povidone-iodine						Washed with 10% povidone-iodine				
Irrigation duration	30s	60s	180s	30s	60s	180s	30s	60s	180s	30s	60s	180s	30s	60s	180s
Sample 1	TNTC	TNTC	TNTC	NG	NG	NG	NG	NG	NG	NG	NG	NG	NG	NG	NG
Sample 2							NG	NG	NG				NG	NG	NG
Sample 3							NG	NG	NG				NG	NG	NG

**Fig. 4:**
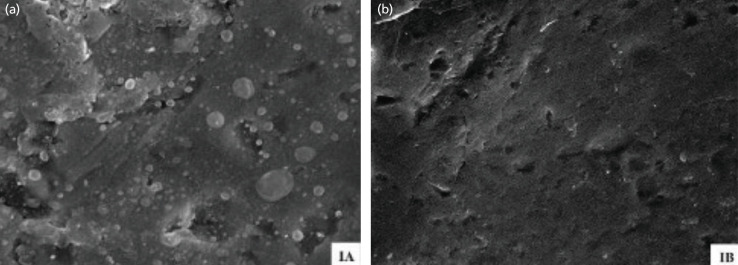
(a) Biofilm was visible, covering the surfaces in a heterogeneous fashion, seen to varying degrees under the scanning electron microscope in group IA (positive control group), (b) while no biofilm was visible in group IB (negative control group).

In group IA, biofilms were visibly present, covering the surfaces in a heterogeneous fashion to varying degrees in the SEM images. This served as our positive control group, where S*. aureus* was cultivated after being washed only with PBS solution (Fig. 4). Conversely, in the other subgroups, there was no growth of S*. aureus* and no presence of biofilms.

**Fig. 5:**
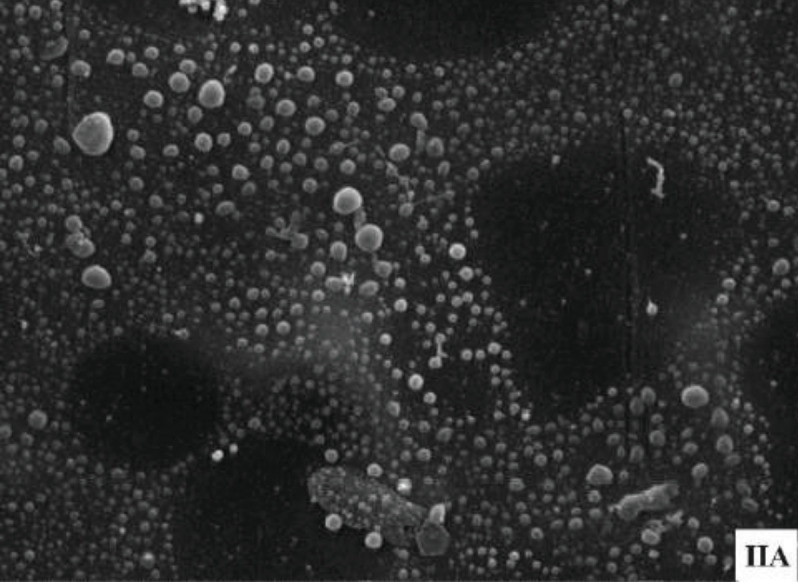
Biofilm was visible under the scanning electron microscope only in group IIA. It also appeared denser compared to before reincubation, as seen in group IA.

In Group II, the SEM results mirrored the findings of the CFU test. In group IIA, biofilms were observed, covering the surfaces in a heterogeneous fashion. The biofilm appeared denser in the reincubation phase compared to the biofilm seen in group IA (Fig. 5).

## Discussion

In clinical practice, orthopaedic implants, especially those related to fractures are commonly made of stainless steel. However, these implants can be complicated by infections. When treating an infected implant, maintaining fracture stability is crucial, not only for achieving fracture union but also for controlling the infection. In certain cases, particularly when the implant is stable and the infection is acute, debridement with retention of the implant is considered as an option. Irrigation with an antiseptic may be used as an adjunct to debridement. The choice of antiseptic includes options such as chlorhexidine gluconate, hydrogen peroxide, and povidone-iodine.

Povidone-iodine has sustained its popularity for decades as an antiseptic for wounds due to its favourable efficacy and tolerability. Several factors contribute to its widespread use, including a broad spectrum of activity, the ability to penetrate biofilms, absence of associated resistance, anti-inflammatory properties, low cytotoxicity, and good tolerability. Importantly, clinical practice has not shown any negative effects on wound healing^21^. In the composition of povidone-iodine, iodine forms a complex with the synthetic carrier polymer povidone, which itself lacks microbicidal activity. In an aqueous medium, free iodine is released into solution from the povidone-iodine complex, establishing an equilibrium^22^. The microbicidal activity of iodine involves inhibiting vital bacterial cellular mechanisms and structures. It also oxidises nucleotides, fatty/amino acids in bacterial cell membranes, and cytosolic enzymes involved in the respiratory chain, leading to denaturation and deactivation^15^. Commercially prepared povidone-iodine solutions or paints typically contain approximately 90% water, 9% povidone, and 1% available iodine. However, the use of povidone-iodine carries some risks for patients, as systemic absorption can occur. Caution should be exercised, especially when using this preparation in high-risk populations, such as severe burn victims and newborns.

In our study, both Group IC and Group IE (with 5% and 10% concentrations of povidone-iodine, respectively) demonstrated successful eradication of *S. aureus* biofilm on stainless steel implants compared to the positive control group. This success was confirmed by the absence of CFU counts and no visible biofilm in SEM. These findings align with a study conducted by Schwechter *et al*^23^, where the effectiveness of various antiseptic solutions, including chlorhexidine gluconate, povidone-iodine, and castile soap, was investigated. The CFU count results indicated that all antiseptics led to lower CFU counts after irrigation, with povidone-iodine ranking second to chlorhexidine gluconate in reducing CFU counts. It is important to note that our study differs as it focuses on methicillin-resistant *S. aureus* biofilm on titanium implants.

We expanded our study to explore the potential for any rebound growth of biofilm after treatment with povidone-iodine. To simulate an in vivo scenario and replicate wound closure, we subjected the implants to a 24-hour reincubation period after submersion in povidone-iodine. Surprisingly, we observed no rebound growth in Group IIC and IIE (reincubation for 24 hours post-treatment with 5% and 10% povidone-iodine, respectively). This contrasts with the findings from the study by Schwechter *et al*^23^, which indicated the presence of rebound growth albeit with smaller CFU counts following povidone-iodine scrub. We hypothesised that this dissimilarity could stem from the differences in implant adhesion between the two microorganisms: methicillin-resistant *S. aureus* (MRSA) versus methicillin-sensitive *S. aureus* (MSSA). It is known that MRSA exhibit higher resistance than MSSA.

As mentioned earlier, this study also investigates the impact of different durations of povidone-iodine submersion in the effectiveness of *S. aureus* biofilm eradication. We discovered that povidone-iodine was successful in eliminating *S. aureus* biofilm even within a short duration of 30 seconds, indicating its rapid action. A study by Kunisada *et al*^24^ explored the efficacy of povidone-iodine against antiseptic-resistant species, including MRSA, Serratia marcescens, *Pseudomonas aeruginosa*, and *Burkholderia cepacia*. They observed that after an exposure time of 30 seconds, no viable cells were detected.

In existing literature, CFU count is commonly employed as a means of validating the efficacy of biofilm eradication. In our study, we incorporated SEM as part of the assessment to confirm the absence of biofilm. SEM allowed us to visualise the morphology of bacteria and biofilm, providing additional evidence that no microorganisms remained after submersion with povidone-iodine. Our findings were consistent across both CFU count and SEM. An intriguing observation emerged in the positive control group, where SEM revealed a denser and more tightly packed morphology of biofilm after 24 hours of reincubation. We speculate that this might be attributed to the evolution of the biofilm into a more complex structure as part of its maturation process.

This study shares the common limitations of *in vitro* research. The biofilm was cultivated in vitro, and therefore, the results may not precisely mirror the characteristics of biofilms *in vivo*. Additionally, the biofilm used in our study was incubated for only one day, potentially limiting its representation of more mature and complex biofilm structures that could yield different outcomes. Furthermore, our investigation focused on a single microorganism and was conducted exclusively on a single type of implant material.

## Conclusion

From our in vitro study, we determined that a concentration of 5% povidone-iodine with a minimum submersion duration of 30 seconds is sufficient to eliminate *S. aureus* biofilm on stainless steel implants. Both CFU count and SEM provided similar results in confirming the absence of biofilm. In addition, SEM enabled visualisation of the biofilm's morphology.

## References

[1] Muller ME (1963). Internal fixation for fresh fractures and for non-union.. Proc R Soc Med.

[2] Harris WH, Sledge CB (1990). Total hip and total knee replacement (1).. N Engl J Med.

[3] Nazhat SN, Young AM, Pratten J (2021). Sterility and Infection. In: Narayan R, ed. Biomedical Materials. 2nd ed.. Springer Cham;.

[4] Zimmerli W, Sendi P (2011). Pathogenesis of implant-associated infection: the role of the host.. Semin Immunopathol.

[5] Jämsen E,, Virta LJ, Hakala M,, Kauppi MJ,, Malmivaara A,, Lehto MU. (2013;). The decline in joint replacement surgery in rheumatoid arthritis is associated with a concomitant increase in the intensity of anti-rheumatic therapy: a nationwide register-based study from 1995 through 2010.. Acta Orthop..

[6] Oliveira WF, Silva PMS, Silva RCS, Silva GMM, Machado G, Coelho LCBB (2018). Staphylococcus aureus and Staphylococcus epidermidis infections on implants.. J Hosp Infect.

[7] Albavera-Gutierrez RR, Espinosa-Ramos MA, Rebolledo-Bello E, Paredes-Herrera FJ, Carballo-Lucero D, Valencia-Ledezma OE (2024). Prevalence of Staphylococcus aureus Infections in the Implantation of Orthopedic Devices in a Third-Level Hospital: An Observational Cohort Study.. Pathogens.

[8] Campoccia D, Montanaro L, Arciola CR (2006). The significance of infection related to orthopedic devices and issues of antibiotic resistance.. Biomaterials.

[9] Kalita SJ, Verma S (2010). Nanocrystalline hydroxyapatite bioceramic using microwave radiation: Synthesis and characterization.. Mater Sci Eng C Mater Biol Appl.

[10] Trobos M, Firdaus R, Svensson Malchau K, Tillander J, Arnellos D, Rolfson O (2022). Genomics of Staphylococcus aureus and Staphylococcus epidermidis from Periprosthetic Joint Infections and Correlation to Clinical Outcome.. Microbiol Spectr.

[11] Zimmerli W, Trampuz A, Ochsner PE. (2004). Prosthetic-joint infections.. N Engl J Med..

[12] Smith DC, Maiman R, Schwechter EM, Kim SJ, Hirsh DM (2015). Optimal Irrigation and Debridement of Infected Total Joint Implants with Chlorhexidine Gluconate.. J Arthroplasty.

[13] Anglen JO, Apostoles S, Christensen G, Gainor B (1994). The efficacy of various irrigation solutions in removing slime-producing Staphylococcus.. J Orthop Trauma.

[14] Moussa FW, Gainor BJ, Anglen JO, Christensen G, Simpson WA (1996). Disinfecting agents for removing adherent bacteria from orthopaedic hardware.. Clin Orthop Relat Res.

[15] Hosaka Y, Saito A, Maeda R, Fukaya C, Morikawa S, Makino A (2012). Antibacterial activity of povidone-iodine against an artificial biofilm of Porphyromonas gingivalis and Fusobacterium nucleatum.. Arch Oral Biol.

[16] Coles VE, Puri L, Bhandari M, Wood TJ, Burrows LL (2024). The effects of chlorhexidine, povidone-iodine and vancomycin on growth and biofilms of pathogens that cause prosthetic joint infections: an in-vitro model.. J Hosp Infect.

[17] Parker DM, Koch JA, Gish CG, Brothers KM, Li W, Gilbertie J (2023). Hydrogen Peroxide, Povidone-Iodine and Chlorhexidine Fail to Eradicate Staphylococcus aureus Biofilm from Infected Implant Materials.. Life (Basel).

[18] Shohat N, Goh GS, Harrer SL, Brown S (2022). Dilute Povidone-Iodine Irrigation Reduces the Rate of Periprosthetic Joint Infection Following Hip and Knee Arthroplasty: An Analysis of 31,331 Cases.. J Arthroplasty.

[19] Goswami K, Austin MS (2019). Intraoperative povidone-iodine irrigation for infection prevention.. Arthroplast Today.

[20] Barreto R, Barrois B, Lambert J, Malhotra-Kumar S, Santos-Fernandes V, Monstrey S (2020). Addressing the challenges in antisepsis: focus on povidone iodine.. Int J Antimicrob Agents.

[21] Bigliardi PL, Alsagoff SAL, El-Kafrawi HY, Pyon JK, Wa CTC, Villa MA (2017). Povidone iodine in wound healing: A review of current concepts and practices.. Int J Surg.

[22] Rackur H (1985). New aspects of mechanism of action of povidone-iodine.. J Hosp Infect.

[23] Schwechter EM, Folk D, Varshney AK, Fries BC, Kim SJ, Hirsh DM (2011). Optimal irrigation and debridement of infected joint implants: an in vitro methicillin-resistant Staphylococcus aureus biofilm model.. J Arthroplasty.

[24] Kunisada T, Yamada K, Oda S, Hara O (1997). Investigation on the efficacy of povidone-iodine against antiseptic-resistant species.. Dermatology.

